# Pan-Cancer Gene Analysis of m6A Modification and Immune Infiltration in Uterine Corpus Endometrial Carcinoma

**DOI:** 10.1155/2022/6530884

**Published:** 2022-09-26

**Authors:** Bing-fan Xie, Yan Xia, Dan-huan Lin, Bing Lian, Meng-li Zhang, Lu Liu, Chun-Rong Qin

**Affiliations:** The Reproductive Medical Center, Shenzhen Maternity and Child Healthcare Hospital The First School of Clinical Medicine Southern Medical University, Guangzhou 518000, Guangdong Provine, China

## Abstract

**Objective:**

This investigation was to test the potential role of m6A-related long non-coding RNAs (lncRNAs) and immune infiltration as crucial factors in the diagnosis and treatment of uterine corpus endometrial cancer (UCEC).

**Method:**

The UCEC RNA-seq data were downloaded in the Cancer Genome Atlas (TCGA, https://portal.gdc.cancer.gov/). There were 587 samples totally, containing 543 UCEC cases and 35 healthy cases. The clinical information of UCEC cases included survival time, survival status, gender, age, stage, and TMN stage. Twenty-three m6A-related genes were found in published journals. The RNA-seq documents of UCEC were downloaded in the Cancer Genome Atlas (TCGA). The hub gene data of UCEC were downloaded from GEPIA2 database. The different packages of R language were applied to calculate and analyze in this research.

**Results:**

Among 587 cases in our study, we discovered 3039 lncRNAs in the TCGA-UCEC database. After the differential analysis, 23 m6A-associated genetics were screened and twenty-one m6A-associated differential genetics were found. In the end, we obtained 20 m6A-related lncRNAs. LNCTAM34A was considered as a predictive gene through univariate and multivariate Cox regression analysis. In addition to the above, patients with high LNCTAM34A expression had better outcomes than those with low LNCTAM34A expression. The high-risk cohort had greater scores of activated dendritic cells (aDCs), B cells, and T cell regulatory (Tregs) than low-risk cohort; in the meanwhile, high-risk cohort had lower scores of DCs and iDCs. Then, the high-risk cohort displayed greater scores in the immune functions of MHC class I, para-inflammation, and type I IFN response than those of low-risk cohort. Among 27 immune-inducible genes, the level of CD244, KIR3DLI, NRP1, PDCD1LG2, and TNFRSF8 was reduced in UCEC samples and the level of CD27, CD28, CD70, CD80, CD86, HAVCR2, ICOS, IDO1, LAIR1, PDCD1, TIGIT, TNFRSF18, -25, -9, -14, and VTCN1 was increased in UCEC samples.

**Conclusion:**

The key role of M6A-related lncRNAs in immune microenvironment in high-risk patients of UCEC. The patients with strong expression of LNCTAM34A have a good prognosis, and LNCTAM34A can be used as a prognostic gene for UCEC. m6A-related lncRNAs can be used as a potential treatment for UCEC. Our observations can be used as a hypothetical basis for future in vitro and animal experiments.

## 1. Introduction

Uterine corpus endometrial carcinoma (UCEC) is still the most frequently diagnosed disease of the female reproductive system in industrialized economies, accounting for over 83% in endometrial carcinoma (EC) [1, 2]. So, even though surgical intervention alone might help treat many UCEC patients, a massive number of female patients with more aggressive histopathological type of the EC continue to have a worse prognosis. At present, there are two main problems in clinic, one of which is the problem of diagnosis, and the other is the difficulty of treatment. The difficulty of diagnosis reflects how to identify risk factors on the basis of biopsy. The difficulty of treatment lies in how to determine the type of adjuvant therapy by using the scope of adjuvant surgery and postoperative evaluation of risk biomarkers [3].

There is accumulating proof that genome destabilization, activation of oncogenes, abnormal methylated modifications, altered epigenetics, unusual expression of microRNAs, and changes in switching signaling processes are the key players and contribute to the malignant pathogenesis [4–7]. Methylation is an important epigenetic modification that is linked to oncogenesis [8]. In eukaryotes, N6-methyladenine (m6A) has been one of the most widely known methylation patterns [9]. According to recent findings, m6A modifications may behave as biologically relevant epigenetic indicators in biological mechanisms [10, 11].

m6A is one of the commonest RNAs modified in all eukaryotes, regulating RNA properties like the ability to splice and encode proteins through writers, erasers, and readers [12]. The m6A modification can be found in a variety of RNA and DNA molecules [13] and even alter the fate of carcinoma cells [14]. According to epi-transcriptomic research, m6A modifications in the transcribed various gene mutations like MYC are engaged in malignant growth and metastatic spread [15]. The abnormal interaction with both writers and erasers, which results from changes in their interpretation, has been connected to the pathological process of carcinoma [16]. However, the m6A genome of endometrial cancer remains unclear in terms of pan-cancer variations.

Despite increasing evidence that m6A factors influence the presence and progression of tumors, the involvement of the role of m6A modified lncRNAs in UCEC remains unknown [17]. A number of lncRNA studies have confirmed aberrant lncRNAs in UCEC specimens, and some of these lncRNAs networks may be important in the development of UCEC [18]. For example, LNC04080 has been observed to be significantly upregulated in EC tissue with involvement in EC carcinogenesis [18]. In addition, LNCTAM34A has been previously suggested to promote glioma proliferation and migration [19].

Based on the above, we undertook a pan-cancer genomic analysis of m6A regulatory factors and immune infiltration in this investigation to obtain a complete overview in EC, which would support in finding new prospects for carcinoma early diagnosis, therapies, and preventative measures.

## 2. Methods

### 2.1. Data Download

The UCEC RNA-seq data were downloaded in the Cancer Genome Atlas (TCGA, https://portal.gdc.cancer.gov/). There were 587 samples totally, containing 543 UCEC cases and 35 healthy cases. The clinical information of UCEC cases included survival time, survival status, gender, age, stage, and TMN stage. Twenty-three m6A-related genes were found in published journals [20–22].

### 2.2. Screening of Prognostic Genes

The package of org.Hs.eg.db was used by R language. 60488 gene IDs and 34446 symbols were obtained after conversion. The same profiles of gene symbol expression were merged by maximum value. The package of normalizeBetweenArrays was used by limma. 24064 differential genes (adj. P. Val<0.5) were found between UCEC and normal cohorts. The lncRNAs were defined using lncRNA annotation file of the GENCODE website. 3039 lncRNAs were totally confirmed from TCGA-UCEC cohort. To assess the relationship between lncRNAs and 21 m6A-associated differential genomics, 20 lncRNAs were found by using the “rcorr” function in the Hmisc package with abs (cor-Filter) > 0.4 and *P* < 0.05. The differential genes of M6A were drawn by ggplot2 package of R language, and the box diagram of m6A-related lncRNA was drawn by ggpubr package of R language. The igraph package of R language is used to draw the lncRNA-related network of m6A.

### 2.3. Construction of lncRNA Risk Score Model

The construction of lncRNA risk score model was combined with the m6A correlation map of 20 lncRNA genes. The clinical data of patients including survival time and status were used for survival analysis by R language and univariate Cox regression. The prognostic genes were obtained from multivariate Cox regression analysis (*P* < 0.05). By the regression coefficients of the screened prognostic genes and their expression profiles, the risk scales were computed through the regression coefficients of screened prognostic genes and their expression profiles:

(1) $$\begin{array}{c} \text{Risk Score}=\sum \text{coef}\left(\text{IncRNAn}\right)\times \text{expr}\left(\text{IncRNAn}\right). \end{array}$$

Note: coef(lncRNAn) represents the regression coefficient of lncRNAs and is obtained from multifactor Cox regression; expr (lncRNAn) represents the expression profile of lncRNAs.

Based on this formula, the risk score of each sample was derived [23]. All UCEC samples containing prognostic information were classified as high risk and low risk in accordance with the median risk score.

### 2.4. The Verification of UCEC Risk Score Model

The heat maps, sample risk status maps, and sample survival status maps were set up. The packages of pheatmap, ggplot2, survminer, and survivalROC were applied.

### 2.5. Investigation of Immune Infiltrating Cells and Functions

TCGA-UCEC expression profiling was conducted applying immunedeconv package of R language. The immunedeconv package enabled a variety of immune infiltration analysis methods, including quantiseq, timer, cibersort, cibersort_abs, mcp_counter, xcell, and epic. Xlsx was obtained from https://onlinelibrary.wiley.com/doi/ftr/10.1002/jcp.29842, containing 16 immune cells and 13 immune-related functions [24]. The ggpubr package of R language is used to draw ggboxmap function box diagrams and to distinguish between high risk and low risk. The gsva package of R language is used to calculate the ssGSEA score of the expression profile of the sample.

### 2.6. Diagnostic Assessment of Hub Gene

The expression of LNCTAM34A gene in TCGA-UCEC was extracted and the expression differences of LNCTAM34A gene were plotted in box plots using the ggpubr package in R language. GEPIA2 database (http://gepia2.cancer-pku.cn/) was utilized to further confirm the difference in its expression in cancer and normal tissues. The specimens of UCEC were classified as high- and low-expression groups based on the median LNCTAM34A levels. The survival time and survival status of patients with UCEC were analyzed by the Kaplan–Meier curve.

## 3. Results

### 3.1. The Screening for m6A-Associated lncRNAs

**Figure 1:**
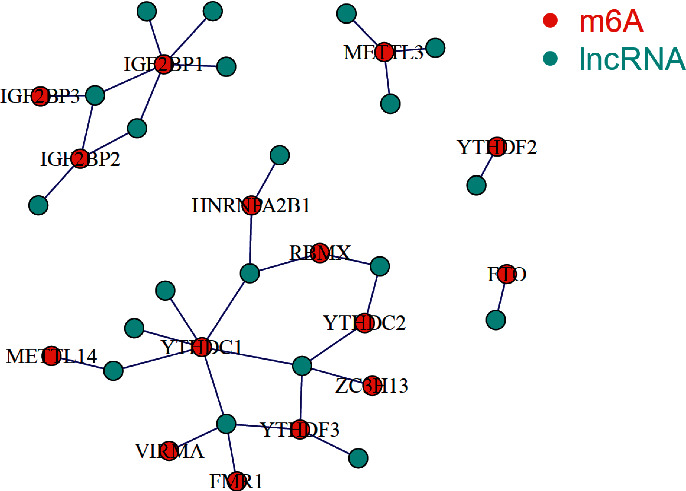
The co-expression networks indicated m6A-associated lncRNAs in UCEC. The red dots were m6A RNA methylated factors and the blue dots were m6A-associated lncRNAs.

**Figure 2:**
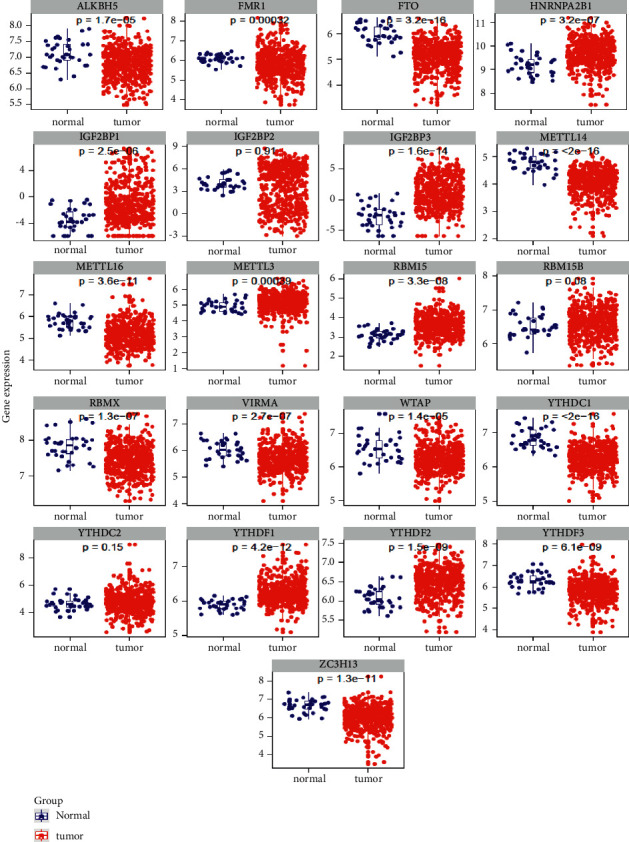
Among the 21 differential genes associated with m6A, there were 9 genes upregulated in UCEC (including IGF2BP3, YTHDF1, YTHDF2, RBM15, HNRNPA2B1, IGF2BP1, METTL3, RBM15B, and YTHDC2) and 12 genes downregulated (including YTHDC1, METTL14, FTO, ZC3H13, METTL16, RBMX, YTHDF3, VIRMA, WTAP, ALKBH5, FMR1, and IGF2BP2).

**Figure 3:**
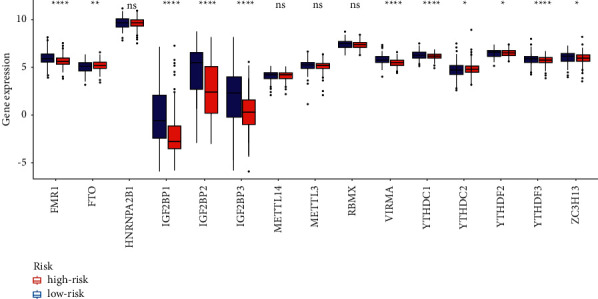
The expressed levels were compared in the high-risk and low-risk groups among the 15 m6A-lncRNAs in correlation network. ^∗^*P* < 0.05.

In this study, a total of 587 samples were obtained from TCGA datasets, including 543 tumor samples and 35 normal samples. The medical characteristics of UCEC patients were analyzed by the above TCGA dataset. Based on the results of the differential analysis, 23 m6A-related genes were screened and 21 m6A-related differential genes were obtained. lncRNAs that were linked to the m6A methylation regulators (abs (cor-Filter) >0.4 and *P* < 0.05) were defined as m6A-associated lncRNAs. The m6A-lncRNA correlation networks are displayed in Figure 1. Among 21 m6A-related differential genes, the upregulated genes were IGF2BP3, YTHDF1, YTHDF2, RBM15, HNRNPA2B1, IGF2BP1, METTL3, RBM15B, and YTHDC2 in UCEC. In addition, the downregulated genes were YTHDC1, METTL14, FTO, ZC3H13, METTL16, RBMX, YTHDF3, VIRMA, WTAP, ALKBH5, FMR1, and IGF2BP2 in UCEC. The details are shown in Figure 2. Among the 15 m6A-lncRNAs in correlation network, the expressed levels were compared in the high-risk and low-risk groups (Figure 3). In the high-risk group, the levels of FMR1, IGF2BP1, IGF2BP2, IGF2BP3, VIRMA, YTHDC1, YTHDF3, and ZC3H13 expressions were obviously higher than those in low-risk group (Figure 3). On the other hand, the levels of FTO, YTHDC2, and YTHDF2 in low-risk group were significantly reduced (Figure 3).

### 3.2. Construction of the UCEC Risk Model

**Figure 4:**
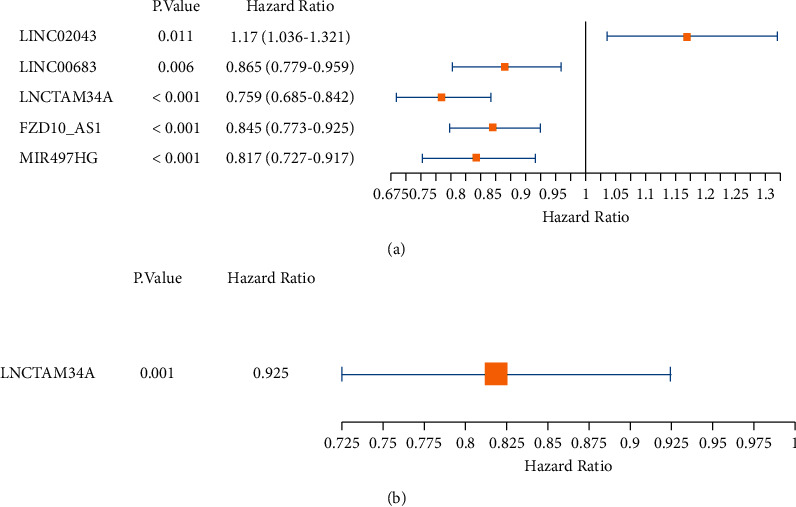
The univariate and multivariate Cox regression analysis was demonstrating five m6A-related lncRNAs.

Five lncRNA prognostic genes associated with disease risk were totally obtained by univariate Cox regression analysis, which were LINC02043, LINC00683, LNCTAM34A, FZD10-AS1, and MIR497HG (Figure 4(a), *P* < 0.05). Multivariate survival analysis using Cox's regression model was applied to support the significant predictive valuation of the five genomic mentioned at the first step. LNCTAM34A was considered as a prognostic gene (hazard ratio = 0.925).

### 3.3. The Verification of UCEC Risk Model

**Figure 5:**
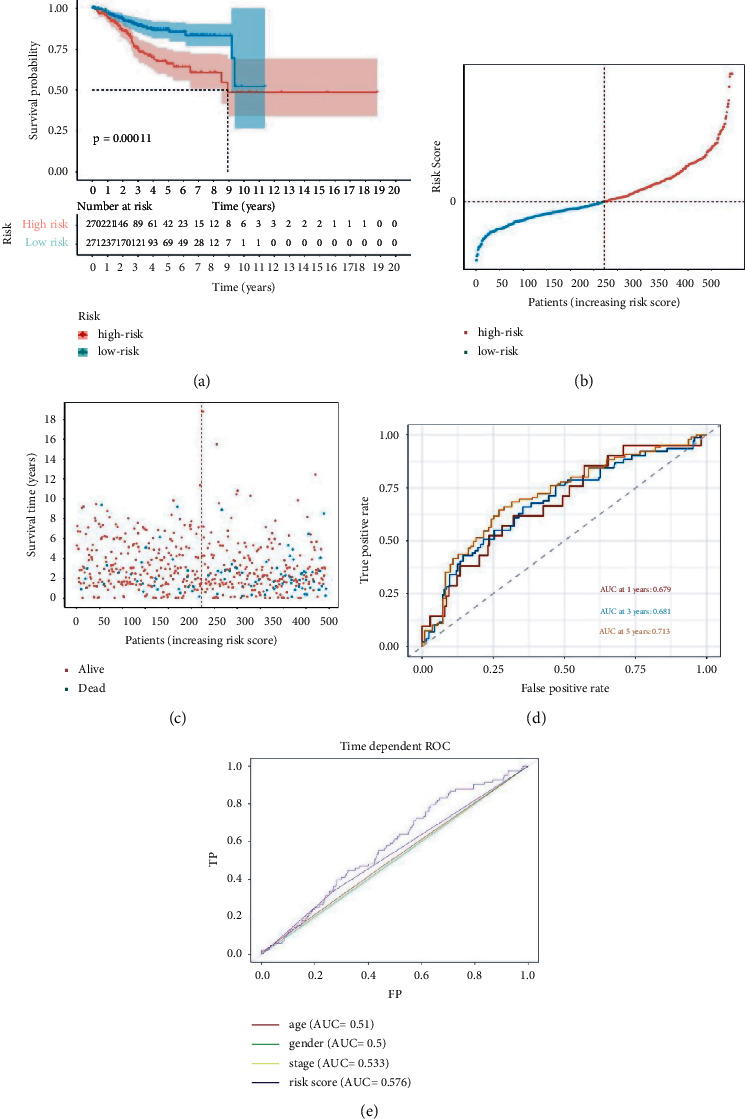
(a) The Kaplan–Meier curves. (b) Distributions of risk scores. (c) Survival status. (d) The AUCs at one, three, and five years to predict OS. (e) The AUCs of OS in the age, gender, tumor stage, and risk score.

The Kaplan–Meier curves displayed that the cases in the high-risk cohort had a poor overall survival (OS) than those in the low-risk cohort (Figure 5(a)). The distributions of risk scores and survival status are shown in Figures 5(b) and 5(c), respectively. The ROC curves showed that high-risk cases were able to predict the OS in UCEC patient cohort. Moreover, the AUCs of OS at one, three, and five years were 0.679, 0.681, and 0.713 separately (Figure 5(d)). The AUCs of OS in the age, gender, tumor stage, and risk score were 0.510, 0.500, 0.533, and 0.576 (Figure 5(e)).

### 3.4. Immune Infiltration Analysis between the High and Low-Risk Groups

**Figure 6:**
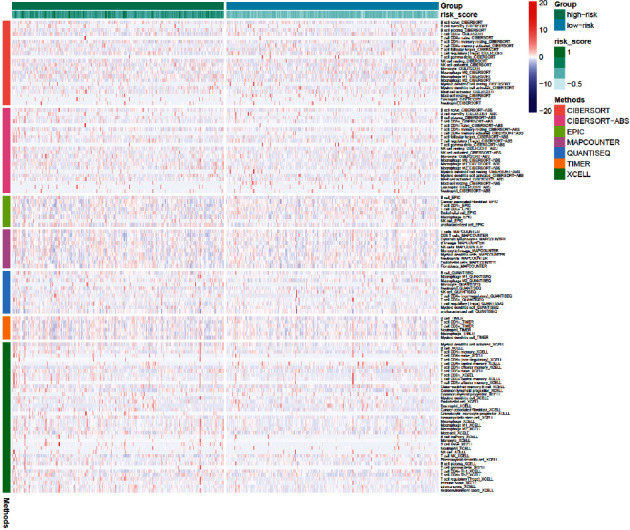
The heatmap displayed seven immuno-infiltration algorithms. The row of the heat map represents the different algorithms of immune cells; the column represents the sample. The rows were classified into seven types of immune infiltration and the columns were classified by high risk, low risk, and risk score.

**Figure 7:**
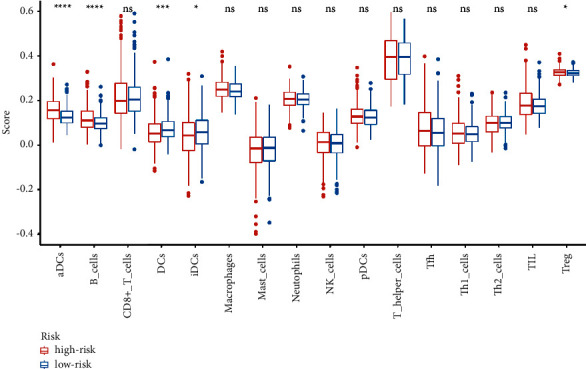
The comparison of immuno-infiltration scores was shown between high-risk and low-risk groups. ^∗^*P* < 0.05, ^∗∗∗^*P* < 0.001, and ^∗∗∗∗^*P* < 0.0001; ns indicated not statistically significant.

**Figure 8:**
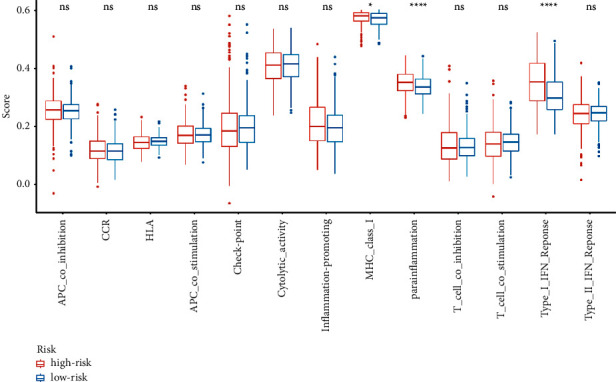
The comparison of immune functional scores was shown between high-risk and low-risk groups. ^∗^*P* < 0.05, ^∗∗∗^*P* < 0.001, and ^∗∗∗∗^*P* < 0.0001; ns indicated not statistically significant.

**Figure 9:**
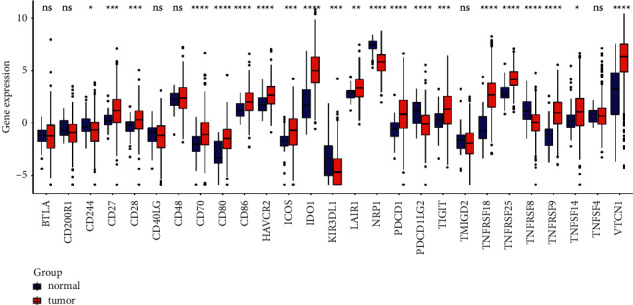
The levels of 27 immune-inducible genes were compared between normal and UCEC samples. ^∗^*P* < 0.05, ^∗∗^*P* < 0.01, and so on; ns indicated not statistically significant.

Differential expression immunologic response-related genotypes were evaluated between the low and high-risk groups to further investigate the possible underlying pathways of the predictive approach and its effectiveness in forecasting the effectivity of immunotherapeutic approaches. The results obtained from seven immuno-infiltration algorithms are displayed in Figure 6. The high and low-risk cohorts were classified via ssGSEA analysis. The infiltration of 16 subtypes of immune cells in high and low-risk cohorts is exhibited in Figure 7. According to Figure 7, high-risk cohort had greater scores of activated dendritic cells (DCs), B cells, and T cell regulatory (Tregs) than low-risk group; in the meanwhile, high-risk group had lower scores of DCs and iDCs. Then, the high-risk group displayed greater scores in the immune functions of MHC class I, para-inflammation, and type I interferon (IFN) response than those of low-risk cohort (Figure 8). Among 27 immune-inducible genes, the levels of CD244, KIR3DLI, NRP1, PDCD1LG2, and TNFRSF8 were reduced in UCEC samples and the levels of CD27, CD28, CD70, CD80, CD86, HAVCR2, ICOS, IDO1, LAIR1, PDCD1, TIGIT, TNFRSF18, -25, -9, -14, and VTCN1 were increased in UCEC samples (Figure 9).

### 3.5. Diagnostic Assessment of Hub Gene

**Figure 10:**
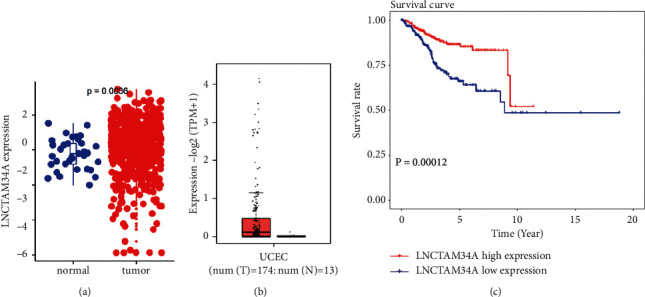
(a) The level of LNCTAM34A was expressed between normal and UCEC patients in TCGA database. (b) LNCTAM34A-related m6A genes were exhibited between normal and UCEC patients in GEPIA2 database. (c) The Kaplan–Meier curves were shown between LNCTAM34A high-expression and low-expression groups.

According to the TCGA-UCEC data, we found that LNCTAM34A was expressed more strongly in normal specimens than that of UCEC samples (*P* = 0.0036, Figure 10(a)). Moreover, GEPIA2 database (http://gepia2.cancer-pku.cn/) was utilized to further confirm the difference in its expression in cancer and normal tissues. Differential expression of m6A related to LNCTAM34A was remarkably stronger in UCEC patients than that in normal samples (*P* < 0.05, Figure 10(b)). The Kaplan–Meier curves suggested that the patients of the LNCTAM34A high-expression cohort had better OS than those in LNCTAM34A low-expression group (*P* = 0.00012, Figure 10(c)).

## 4. Discussion

The emergence and advancement of UCEC is a multifactorial and complicated process [25]. The genetic information is translated into an extremely complicated RNA network for the most part. On the other hand, only 1% to 2% of transcripts are translated into proteins [26]. Therefore, RNA post-transcriptional regulation is particularly important because this post-transcriptional regulation can regulate the activity of tumor RNA transcription, thus changing the function and outcome of tumor cells [27]. lncRNAs are the transcription factors that are commonly regarded as transcribed far over 200 nucleotides which are not translated into proteins. Former research has linked the dysfunctional particular lncRNAs to the emergence and progression of malignancies. This research used pan-cancer genomic evaluation to profile m6A modification and expressed patterns of lncRNAs in order to gain a better insight into the underlying involvement of lncRNAs in human UCEC.

Among 587 cases in our study, we discovered 3039 lncRNAs in the TCGA-UCEC database. After the differential analysis, 23 m6A-related genes were screened and 21 m6A-related differential genes were found. In the end, we obtained 20 m6A-related lncRNAs. LNCTAM34A was considered as a prognostic gene through univariate and multivariate Cox regression analysis. Moreover, the expression of LNCTAM34A was significantly stronger in normal cohort than that in UCEC cohort based on the TCGA-UCEC database. The patients of the LNCTAM34A high-expression cohort had better OS than those in LNCTAM34A low-expression group. LNCTAM34A was initially identified as an anti-sense RNA capable of modulating the cancerous inhibitor microRNA-34a in a variety of human malignancies [28]. Our results are consistent with data from previous studies, and in addition, our study is able to be interpreted through the pathways found [29]. Previous investigation found that LNCTAM34A-mediated upregulation of microRNA-34a levels was adequate to motivate the proper responses of cells to such stressful stimuli [29]. When exposed to multiple types of cellular strain, the collaboration of TP53 with other variables can initiate transcription at the microRNA-34a site, contributing to the formation of LNCTAM34A and miR34a [30, 31]. It has been reported that low-level expression of LNCTAM34A has been linked to a lower survival rate than that of high-level expression of LNCTAM34A in cancer patients [29]. In addition, worse outcomes of UCEC patients were discovered when occurence of TP53 mutation, and low levels of microRNA and LNCTAM34A. Nevertheless, other studies have shown that LNCTAM34A is seen as a malignant promoter in glioma [19]. When LNCTAM34A was knocked out, glioma cell proliferation was inhibited, migration was diminished, and EMT rates were relatively low [19]. This contradictory conclusion may be due to the direct heterogeneity of the different types of neoplasms [32]. Therefore, further exploration will need to be undertaken to explore the mechanisms of lncTAM34a regulation in UCEC.

The treatment of malignant tumours has been challenged by the properties of cancer relapse and metastasis [33]. Immunotherapy is one of the potential therapies for patients with UCEC, so m6A associated with regulating immune infiltration may have an effect on EC immune checkpoint inhibitors [34]. In our study, high-risk group had greater scores of activated dendritic cells (aDCs), B cells, and T cell regulatory (Tregs) than low-risk group; in the meanwhile, high-risk group had lower scores of DCs and iDCs. Then, the high-risk group displayed greater scores in the immune functions of MHC class I, para-inflammation, and type I IFN response than those in the low-risk group. Among 27 immune-inducible genes, the levels of CD244, KIR3DLI, NRP1, PDCD1LG2, and TNFRSF8 were reduced in UCEC samples and the levels of CD27, CD28, CD70, CD80, CD86, HAVCR2, ICOS, IDO1, LAIR1, PDCD1, TIGIT, TNFRSF18, -25, -9, -14, and VTCN1 were increased in UCEC samples. Dendritic cells (DCs) have specific tumour antigens capable of inducing apoptosis in cancer cells, as well as nucleotide genes encoding pathogen-associated molecular patterns to trigger an immune response [35]. In the complex tumor microenvironment, B cells have diversity, and a variety of B cells regulate the occurrence and development of tumors. In the study of tumor invasion, B cells play an anti-tumor role by mediating T-cell immune function [36]. The findings indicated that immune-related genes exhibited positive correlation to the tumor infiltration of B cells and DCs in EC [37]. The histocompatibility complex (MHC) class I compound is a membrane-bound protein complex that is demonstrated on multinucleate human cells. MHC class I introduces intracellular signaling pieces to T-lymphocytes and initiates an excitation cascade when these cells recognize neoantigen. MHC class I loss by cancerous cells reduces tumor neoantigen demonstration to the immune response and thus reflects a potential mechanism of potential therapeutic tolerance even in malignancies that emerge to be excellent candidates for checkpoint suppression [38].

There are still a few drawbacks to in silico research. The expression patterns and diagnostic features used in this study were obtained from online databases with limited data, and the findings were not verified experimentally. We did, however, undertook multidimensional confirmation across several online databases, which provides strong backing for the relationships with both crucial biological markers recognized in our evaluation.

In conclusion, m6A-associated lncRNAs play a key role in the immune microenvironment of high-risk UCEC individuals. The patients with strong expression of LNCTAM34A have a good prognosis, and LNCTAM34A can be used as a prognostic gene for UCEC. m6A-related lncRNAs can be used as a potential treatment for UCEC. Our observations can be used as a hypothetical basis for future in vitro and animal experiments.

## Data Availability

The datasets generated during analysed are not publicly available but are available from the corresponding author on reasonable request.
